# Effectiveness of Maintenance Immunosuppression Therapies in a Matched-Pair Analysis Cohort of 16 Years of Renal Transplant in the Brazilian National Health System

**DOI:** 10.3390/ijerph17061974

**Published:** 2020-03-17

**Authors:** Rosângela Maria Gomes, Wallace Breno Barbosa, Brian Godman, Juliana de Oliveira Costa, Nélio Gomes Ribeiro Junior, Charles Simão Filho, Mariângela Leal Cherchiglia, Francisco de Assis Acurcio, Augusto Afonso Guerra Júnior

**Affiliations:** 1 Department of Social Pharmacy, College of Pharmacy, Federal University of Minas Gerais, Belo Horizonte 31270-901, Brazil; 2 SUS Collaborating Centre—Technology Assessment & Excellence in Health, College of Pharmacy, Federal University of Minas Gerais, Belo Horizonte 31270-901, Brazil; 3 Programa de Pós-graduação em Medicamentos e Assistência Farmacêutica, Departamento de Farmácia Social, Faculdade de Farmácia, Universidade Federal de Minas Gerais—UFMG. Av. Presidente Antônio Carlos, 6627 Campus Pampulha, Belo Horizonte, MG 31270-901 Brazil; 4 Strathclyde Institute of Pharmacy and Biomedical Sciences, Strathclyde University, Glasgow G4 ORE, UK; 5 Department of Laboratory Medicine, Division of Clinical Pharmacology, Karolinska Institute, Karolinska University Hospital Huddinge, SE-141 86 Stockholm, Sweden; 6 Health Economics Centre, Liverpool University Management School, Chatham Street, Liverpool L69 7ZH, UK; 7 School of Pharmacy, Sefako Makgatho Health Sciences University, Ga-Rankuwa, South Africa; 8 Department of Preventive and Social Medicine, College of Medicine, Federal University of Minas Gerais, Belo Horizonte 31270-901, Brazil; 9 Department of Surgery, College of Medicine, Federal University of Minas Gerais, Belo Horizonte 31270-901, Brazil

**Keywords:** real-world data, real-world evidence, renal transplantation, immunosuppressants, graft survival, effectiveness, tacrolimus, cyclosporine, clinical epidemiology

## Abstract

The maintenance of patients with renal transplant typically involves two or more drugs to prevent rejection and prolong graft survival. The calcineurin inhibitors (CNI) are the most commonly recommended medicines in combinations with others. While immunosuppressive treatment regimens are well established, there is insufficient long-term effectiveness data to help guide future management decisions. The study analyzes the effectiveness of treatment regimens containing CNI after renal transplantation during 16 years of follow-up with real-world data from the Brazilian National Health System (SUS). This was a retrospective study of 2318 SUS patients after renal transplantion. Patients were propensity score-matched (1:1) by sex, age, type and year of transplantation. Kaplan–Meier analysis was used to estimate the cumulative probabilities of survival. A Cox proportional hazard model was used to evaluate factors associated with progression to graft loss. Multivariable analysis, adjusted for diabetes mellitus and race/color, showed a greater risk of graft loss for patients using tacrolimus plus mycophenolate compared to patients treated with cyclosporine plus azathioprine. In conclusion, this Brazilian real-world study, with a long follow-up period using matched analysis for relevant clinical features and the representativeness of the sample, demonstrated improved long-term effectiveness for therapeutic regimens containing cyclosporine plus azathioprine. Consequently, we recommend that protocols and clinical guidelines for renal transplantation should consider the cyclosporine plus azathioprine regimen as a potential first line option, along with others.

## 1. Introduction

Kidney transplantation is considered the optimal choice for the treatment of patients with advanced renal failure due to improved quality of life and life expectancy versus renal dialysis, and it is also the most cost-effective option when compared to dialysis [1,2,3,4,5,6]. The Brazilian National Health System (Sistema Único de Saúde (SUS)) performs more than 95% of all kidney transplants in the country and guarantees access to immunosuppressants for transplant patients without co-payment [7,8].

In line with international guidelines [9,10], the recommended maintenance immunosuppression in Brazil consists of a triple regimen containing a corticosteroid, a calcineurin inhibitor (CNI) (cyclosporine or tacrolimus) and an antiproliferative agent (azathioprine or mycophenolate). Alternatively, either of the two latter drug classes may be replaced by the mammalian target of rapamycin (mTOR), sirolimus or everolimus, depending on the patient’s clinical characteristics [8,11].

Different results regarding the effectiveness and safety among possible immunosuppressive regimes have been reported in multiple studies [7,12,13,14]. With respect to tacrolimus and cyclosporine, considered as key elements of any immunosuppressive regimen, a variety of studies compared their relative effectiveness and safety. A systematic review has not shown differences between tacrolimus and cyclosporine at one and five years of graft survival [15], as well as mortality. However, another systematic review has shown has tacrolimus is superior to cyclosporine in relation to graft loss and overall efficiency [16]. Similar controversies exist in relation to the anti-proliferatives [10] as well as with sirolimus and everolimus [14,16,17]. This illustrates the fact that recommendations for the use of different immunosuppressive regimens are complex and influenced by the type of donor and other clinical factors including prior dialysis length of time, recipient age, and sex [7,18].

Previous real-world studies conducted in Brazil comparing patient outcomes with cyclosporine versus tacrolimus in association with any other immunosuppressant at five [18] and 10-years follow-up [7] showed a clinical benefit favoring cyclosporine. However, considering that the recommended treatment typically consists of a triple regimen, and that time is a fundamental factor in any survival analysis, there is still doubt about which immunosuppressive regimen is the most effective in maintaining renal grafts over a longer time. This is important especially if there are appreciable cost differences between these immunosuppressants as currently seen in Brazil [7]. Consequently, this long-term study was undertaken to deepen our understanding of potential treatment approaches for patients undergoing renal transplantation in Brazil, especially given the heterogeneity of the population and concerns with the results of short-term follow-up studies. As a result, we can provide additional information to the Brazilian Ministry of Health, as well as to health professionals and patients, regarding potential immunosuppressant choices containing either cyclosporine or tacrolimus over 15 years of follow-up.

## 2. Materials and Methods 

### 2.1. Study Design

This non-concurrent open cohort study included all patients who underwent kidney transplantation (living donors or deceased donors) at transplantation centers throughout Brazil. The cohort was developed through deterministic-probabilistic matching of the following administrative databases: SUS Hospital Information System (SIH/SUS), SUS Ambulatory Information System (SIA/SUS) and Mortality Information System (SIM), similar to previous studies undertaken in Brazil [7,19,20]. Notification regarding mortality (SIM) is mandatory in Brazil, and immunosuppressive treatment is dispensed monthly for patients in SUS (SIA/SUS) without any co-payment. Dialysis is recorded in the same way as immunosuppressive therapy.

The study cohort consisted of SUS patients who underwent kidney transplants and received immunosuppressive regimens containing either cyclosporine (Anatomical Therapeutic Chemical (ATC) code: L04AD01) or tacrolimus (L04AD02) between 1 January 2000 and 31 December 2014. Patients were subsequently followed up from 1 January 2001 to 31 December 2015. The entry period was established to ensure a minimum follow-up of 12 months. The date of entry into the cohort corresponded to the date of the transplant registered in SIH/SUS. Patients younger than 18 years and patients who died between the first and the sixth month after transplantation were excluded as this could be related to the surgical procedure and not to a lack of effectiveness of maintenance immunosuppressive drugs, which is in line with previous studies [4,7,18].

The therapeutic regimen was defined as the first regimen the patient was treated with a duration of at least 90 days (intention to treat—ITT). The following regimens were considered, stratified by the use of cyclosporine or tacrolimus: monotherapy, concomitant use of azathioprine (L04AX01), mycophenolate (L04AA06), everolimus (L04AA18) or sirolimus (L04AA10). Any other immunosuppressant regimens used by patients were grouped in the category ‘other regimens’. All patients were considered to have concomitant corticosteroids.

The patients were matched according to propensity score matching 1:1 (one-to-one). This was established by the type of transplant, sex, age in years at the time of transplantation, and year of transplantation, among patients prescribed either cyclosporine or tacrolimus. When more than one individual in any group was a therapeutic candidate for pairing by the four variables, pair allocation was randomly selected.

### 2.2. Event

The event used for the survival analysis was graft loss, defined as the need for dialysis for more than three months without the concomitant use of immunosuppressive medication, re-transplantation or death. The effectiveness of the immunosuppressive medicines was measured by means of graft survival data. The date of the event was defined as the date of return to dialysis, the date of re-transplantation, or the date of death, whichever occurred first. Censorship was characterized as loss of follow-up, adopting the date of the last record referring to immunosuppression, or the end of follow-up (right censoring). Right censoring was defined as not observing the event of interest until the end of study, i.e, any individual that did not have an event until 31 December 2015 was censured.

### 2.3. Statistical Analysis

Descriptive statistical analysis of all variables in this study was performed; that is, the frequency distribution for categorical variables and central tendency for continuous variables. The variables included: (a) the geographic region of the hospital where the transplant was performed; (b) the calendar year of transplantation, categorized as transplants between 2000 to 2003, 2004 to 2007, 2008 to 2011 and 2012 to 2014; (c) the sex of the recipient; (d) the age at the time of transplantation; (e) the skin color/race of the patient; (f) the primary diagnosis of renal disease; (g) the type of transplant received (donor alive or deceased) and (h) the dialysis period prior to renal transplantation.

The cumulative probability of graft survival at 15 years according to the therapeutic regimen was assessed by the Kaplan–Meier estimator, and survival distributions were compared using the log-rank test. Factors influencing graft survival were assessed initially by univariate analysis of each variable and its association with graft loss. Variables with *p* < 0.20 in the univariate analysis and variables considered clinically relevant were included in the multivariable model. The relative risk of progression to the event adjusted for the multivariable model was calculated by the Cox proportional hazards model and was considered as a 95% confidence interval (95% CI). The adequacy of the multivariable model was evaluated by the residue analysis. In order to evaluate the impact of death on graft survival, an analysis of graft loss censored for death, i.e., considering only the return to dialysis as an event, was also performed. In view of comparison between the regimens, we also performed an analysis of patient death with a functional graft.

Statistical analysis was performed using the program R, version 4.3.1 (R Foundation for Statistical Computing, Vienna, Austria) and SPSS, version 17 (SPSS Inc., Chicago, IL, USA) and a significance level of 5% was considered.

### 2.4. Ethical Aspects

The Ethics Committee of the Federal University of Minas Gerais in Brazil approved this study (number 1072253/2015).

## 3. Results

### 3.1. Patient Characteristics and Survival Rates

**Figure 1:**
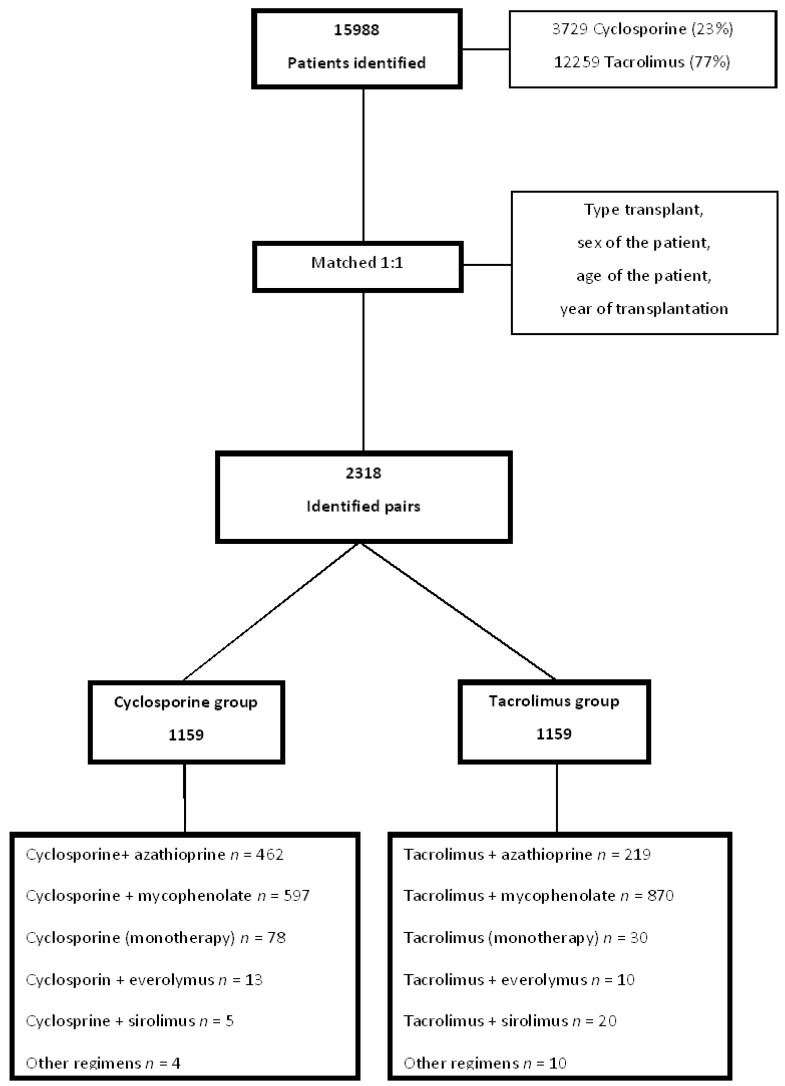
Study flowchart (Brazil, 2000–2015; *n* = 2318).

From January 2000 until December 2015, 3729 (23%) patients were prescribed a cyclosporine based immunosuppressive regimen and 12,259 (77%) patients were prescribed a tacrolimus based regimen. A total of 1159 pairs were combined by type of transplantation, sex, age and year of transplantation between the cyclosporine and tacrolimus groups. The distribution of the number of patients receiving each regimen within the cyclosporine or tacrolimus group is shown in Figure 1.

Of the 2318 patients included in the study, most (63.0%) were prescribed mycophenolate in combination with a CNI, followed by azathioprine (29.0%). Only 3% of the study population were prescribed regimens with sirolimus (1.3%) or everolimus (1.7%). In the tacrolimus group, the majority (75.0%) of the patients were prescribed tacrolimus plus mycophenolate in combination, followed by a tacrolimus plus azathioprine combination (18.8%). In the cyclosporine group, the cyclosporine plus mycophenolate combination was used by half the patients (50.8%) and the cyclosporine plus azathioprine combination by 40.0% of the study patients. The other regimens represented less than 10.0% of patients in both groups (Figure 1).

Most of the patients were male (63.2%), who declared themselves to be white/caucasian (55.0%) and with a median age of 43 years. The main etiology of chronic renal failure was hypertension/cardiovascular disease (19.2%). Most transplants occurred in the southeast region of Brazil (60.9%), followed by the southern region (23.3%). The most common type of transplantation was from a living donor (63.7%) and the median dialysis time before a transplant was 26 months (interquartile interval: 13 to 53 months). During the follow-up, there were 421 graft losses (18.2%) which included graft losses due to death (13.8% of the population), return to dialysis for more than three months (4.0%) and re-transplantation (0.4%). There was also 1897 (81.8%) censures (Table 1).

The effectiveness of the different therapeutic regimens is shown in Table 2. Of the 421 (18.2%) graft losses that occurred, 61% were related to patients who were prescribed a mycophenolate associated regimen, 35.2% of whom were prescribed a mycophenolate plus tacrolimus combination and 25.8% mycophenolate plus cyclosporine. The cyclosporine regimen in combination with azathioprine demonstrated a 25.0% protection in the univariate analysis (*p* = 0.04). The other combinations did not present statistically noticeable differences. Patients who were prescribed the cyclosporine plus azathioprine regimen had a 54.0% survival (95% CI = 43.9 to 66.4) at 15 years of follow-up, whereas patients who were prescribed tacrolimus plus azathioprine had a survival rate of 37.3% (95% CI = 21.5 to 64.6). Mycophenolate regimens showed similar survival rates (Table 2).

Patient graft survival in the paired cohort was 50.1% (95% CI = 43.0 to 58.3) at the end of 15 years of follow-up. Individuals in the cyclosporine group had a graft survival of 52.1% (95% CI = 44.7 to 60.8) at the end of 15 years follow-up, and patients in the tacrolimus group had a survival of 47.5% (95% CI = 36.1 to 62.7) (Table 3). The annual graft survival probabilities observed for each group over the 15-year period are presented in Table 3. 

### 3.2. Factors Associated with Graft Loss

#### 3.2.1. Univariate Analysis

Univariate analysis indicated an increased risk of graft loss for each additional year of age of the recipient (HR = 1.03 (95% CI = 1.02 to 1.03)), an increased risk of graft loss among patients who had a dialysis time of more than 26 months and among those who were transplanted at an earlier time period between 2000 and 2003 (HR = 1.49 (95% CI = 1.21 to 1.83)).

**Figure 2:**
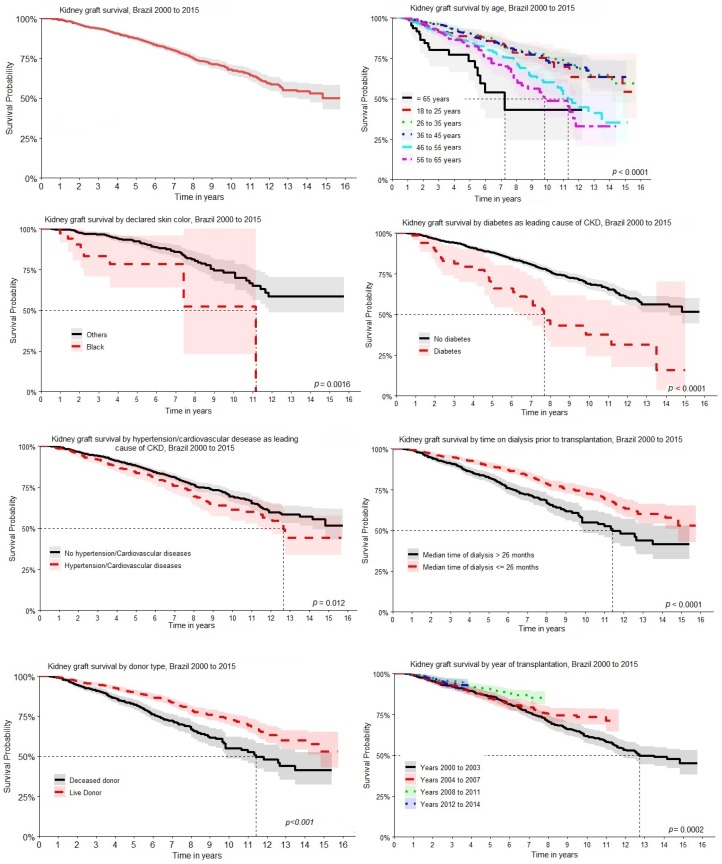
Kaplan–Meier graft survival estimates for 15 years after renal transplantation according to age of recipient, patient skin race/color, chronic kidney disease (CKD) primary diagnosis, donor type, dialysis length of time before transplant, and year of transplantation.

Patients who received a deceased donor organ also had a higher risk of graft loss (HR = 1.77 (95% CI = 1.46 to 2.16)), and similarly, those with diabetes (HR = 1.49 (95% CI = 1.24 to 1.80)) or hypertension/cardiovascular disease (HR = 1.32 (95% CI = 1.06 to 1.66)) as the main cause of chronic kidney disease (CKD). Patients of color/black were also at higher risk of graft loss (HR = 3.09 (95% CI = 1.47 to 6.46)). The graphical representation of the survival rates according to these variables is shown in Figure 2.

#### 3.2.2. Multivariable Analysis

Considering the level of statistical significance used in the univariate analysis (*p* < 0.20) and the relevant epidemiological clinical data, multivariable analyses were performed. Since the race/color of patients has an appreciable impact on graft survival, but information on this variable is missing for a large proportion of the study population (Table 1), two additional multivariable models were considered.

A multivariable analysis including all patients revealed that the following variables were associated with a higher risk of graft loss: patients who had a dialysis length of time longer than 26 months before transplantation (HR= 1:53 (95% CI = 1.25 to 1.88)), as well as patients with a diagnosis of diabetes mellitus (HR = 2.55 (95% CI = 1.55 to 3.71)) and hypertension/cardiovascular diseases (HR = 1.28 (95% CI = 1.102 to 1.61)) as the primary cause of CKD (Table 4).

The multivariable analysis also demonstrated that a greater risk of graft loss was associated with patients diagnosed with diabetes mellitus as the main cause of CKD (HR = 3.14 (95% CI = 1.11 to 8.81)), patients who declared their race/color as black (HR = 2.61 (95% CI = 1.13 to 6.02)) and patients prescribed tacrolimus plus mycophenolate (HR = 2.17 (95% CI = 1.02 to 2.41)) compared to a cyclosporine plus azathioprine regimen (Table 5).

Analysis of the residues demonstrated that the multivariable models showed good adequacy according to Schoenfeld, with an average close to zero and no violation of the homoscedasticity assumption.

### 3.3. Sensitivity Analysis

In the death censored analysis, the overall survival at 15 years was 78.2% (95% CI = 67.4 to 90.6). Patients in the cyclosporine group had a survival of 87.1% (95% CI = 80.7 to 94.1) while those in the tacrolimus group had a survival of 70.3% (95% CI = 52.7 to 93.9) (*p* = 0.04). Regarding the therapeutic regimens, it was observed that patients prescribed tacrolimus plus mycophenolate had a higher risk of graft loss (HR = 1.82, *p* = 0.04), alongside those prescribed tacrolimus plus azathioprine (HR = 1.35, *p* = 0.45) and cyclosporine plus mycophenolate (HR = 1.21, *p* = 0.57) compared with patients who were prescribed cyclosporine plus azathioprine. In the ‘patient death with a functional graft’ analysis, no regimen showed a greater advantage than cyclosporine plus azathioprine. Cyclosporine plus mycophenolate had a higher risk of graft loss (HR = 1.38, *p* = 0.04) compared with cyclosporine plus azathioprine, and the different regimens with tacrolimus showed no statistically noticeable difference (tacrolimus + mycophenolate HR = 0.99, *p* = 0.84; tacrolimus + azathioprine HR = 1.20, *p* = 0.35).

## 4. Discussion

After fifteen years of follow-up, overall renal graft survival was 50.1%. Patients who were prescribed cyclosporine plus azathioprine had a higher Kaplan–Meier survival rate over the 15 years compared to patients using tacrolimus plus azathioprine (54.0% vs 37.3%) (Table 2). Overall, comparing cyclosporine plus azathioprine regimens with all other regimens, our model, adjusted for race and diabetes mellitus, showed superiority for this regimen versus tacrolimus plus mycophenolate, with no differences between cyclosporine and the other regimens (Table 5). Adjusting for diabetes is important since previous studies have reported that patients with diabetes obtain worse post-transplant results [7,18]. In addition, diabetes is usually related to other cardiovascular factors associated with a worse prognosis [21,22]. These findings are similar to other published studies and build on our earlier studies [7,18,23,24].

This compares with Vacher-Coponat et al. (2012) who found that a tacrolimus plus mycophenolate combinaton is not more efficient than a cyclosporine plus azathioprine combination [25]. The cyclosporine plus mycophenolate regimen, which was the second most prescribed regimen in our cohort (26.0%), had no significant associated benefit; however, in our final model, this regimen showed a greater graft loss trend when compared with cyclosporine plus azatioprine (Table 5).

Goldfarb-Rumyantzev et al. [24] reported that a cyclosporine plus mycophenolate regimen is associated with a lower risk for graft failure compared with tacrolimus plus mycophenolate, which was comparable to a cyclosporine plus azathioprine regimen, contrasting with the findings of our study. Wagner et al. in their systematic review reported a 22% reduction in the risk of graft loss due to death (Relative risk (RR )= 0.78 (95% CI = 0.62 to 0.99)) for patients who were treated with mycophenolate versus azathioprine in combination with a CNI [10]. We are not sure of the reasons behind the differences in the findings between the studies; however, this may be related to the type of study and the limitations inherent to each type of study. In addition, we are aware that black patients need higher doses of tacrolimus to achieve therapeutic levels due to specific polymorphisms of CYP3A5. This could, in part, explain the superiority of cyclosporine found in our study [26,27,28], although we were not able to evaluate dosage in this study.

In our study, a more advanced age was associated with worse survival, similar to previous studies [7,18,29,30]. Among the clinical variables, a longer dialysis time before transplant had a negative influence on graft survival, again similar to other studies [7,18,21,30,31]. Previous studies have shown that hypertension and cardiovascular disease, nephritis and pyelonephritis, and diabetes mellitus are the main causes of CKD [7,19,32,33]. The primary diagnoses of CKD, including hypertension, diabetes mellitus and nephritis, are also associated with an increased risk of graft loss. However, only the group of patients with a primary diagnosis of diabetes mellitus and hypertension remained in our final model presenting with a risk of increased graft loss. Miscegenation is a particular demographic characteristic of the Brazilian population [34]. Whilst we did not have the race and color records for all patients, since this information only recently became mandatory in the SUS information systems [35], patients who declared themselves as black presented with a greater risk of loss of graft. These findings are similar to another study conducted in Brazil [36], which also found worse results in patients who declared themselves to be black.

We are aware that tacrolimus has typically replaced cyclosporine and mycophenolate has typically replaced azathioprine in current immunosuppressive regimes [10,37,38]. However, as mentioned, these two immunosuppressants are currently more expensive in Brazil and their effectiveness has been the subject of ongoing debate which has been enhanced by our findings. Other important issues for physicians and authorities to consider when prescribing immunosuppressants include the fact that 30–60% of recipients develop BK viremia with up to 70% losing their transplanted kidney from infection [39,40]. Rationally, one can assume that the stronger the immunosuppressive regimen, the higher the possibility of developing BK viremia. In line with this, Bernnan et al. (2005) [41] evaluated the effect of a CNI with either mycophenolate or azathioprine on the incidence of BK virus infections in renal transplant patients. However, they found that the incidence of BK viremia was equal in patients receiving either a tacrolimus plus mycophenolate or a cyclosporine plus azathioprine regimen. Consequently, it is difficult to say whether a tacrolimus plus mycophenolate combination is currently stronger than a cyclosporine plus azathioprine regimen.

During the last few decades, the introduction of new immunosuppressants with new mechanisms of action has increased the number of options and strategies to avoid rejection, as well as reduce the side effects associated with tacrolimus and cyclosporine therapies. Sirolimus and everolimus (mTOR) are among the newer agents [17]. These immunosuppressants are currently recommended as second treatment line in Brazil [11], which is reflected in their limited use in our study (3.0%). However, there is ongoing controversy regarding the effectiveness of these combinations [13,14,17,42,43,44]. Some clinical trials [45] support the use of everolimus as a standard immunosuppressive drug leading to reduced exposure to CNI; however, this is not universal. A recent systematic review [45] which compared mTOR with other immunosuppressants found that everolimus or sirolimus combined with a CNI prevented kidney transplant failure and rejection as effectively as other immunosuppressants combined with a CNI in short follow-up periods. However, the risk of viral infections (cytomegalovirus and BK) was significantly less with mTOR combined with everolimus. We will be exploring this further in the future in view of the low number of patients prescribed mTOR in our study.

We are aware there are a number of limitations related to the design of this study, and to the source of information; consequently, the results must be evaluated with caution. Our study was retrospective and unfortunately did not provide explanations for all the observed associations. Future studies are needed to confirm identified associations. Administrative records may present incomplete or inconsistent information, inherent in the retrospective nature of the study. In addition, clinical information that potentially affects graft survival, such as acute rejection rates, immunological compatibility, ischemia times, and graft function in the first year after transplantation, was not available in our database. Another limitation is that cyclosporine, by inhibiting enterohepatic circulation of mycophenolate, increases the required dose of mycophenolate. However, blood levels of immunosuppressants and data on adherence to immunosuppressive therapies were also not available. This was despite the Brazilian Guidelines currently recommending monitoring the level of these immunosuppressants, and the SUS funds these procedures. Overall, we believe the difference in graft survival between these regimens can be explained by the difference in nephrotoxicity, associated comorbidity, immunological risk, or different adverse effect profiles.

Despite it is limitations, we believe the long follow-up period, the representativeness of the sample (which included practically the entire population of the country undergoing kidney transplantation), and using immunosuppressants through SUS reflects the real-world scenario in Brazil. Consequently, we believe our findings to be robust, providing guidance not only to key stakeholders in Brazil, but also wider audiences.

## 5. Conclusions

This retrospective, real-world, nationwide study with a long follow-up period revealed better long-term outcomes for cyclosporine plus azathioprine versus other regimens for the maintenance of renal transplants in Brazil. Despite the limitations of the study design and available data, including clinical features, we believe these findings may give guidance to policy makers and prescribers in clinical practice, helping them to choose between several possible combinations as first line treatment. 

We believe real-world studies such as these will become increasingly important in the future to help guide decision making given the short term follow-up in most clinical trials, including those for new immunosuppressants. However, we also suggest that further prospective studies are conducted to add to our findings and to the overall debate regarding the relative merits of the various immunosuppressants. In the meantime, protocols and clinical guidelines for renal transplantation could consider the cyclosporine plus azathioprine regimen as a potential first line option, along with others.

## Figures and Tables

**Table 1:** Demographic characteristics of study patients (Brazil, 2000–2015; *n* = 2318).

Characteristic	All Patients		Cyclosporine Group	Tacrolimus Group
	(*n* = 2318)		(*n* = 1159)	(*n* = 1159)
	*n*	%	%	%
Geographic origin				
Southeast	1370	59.1	25.2	33.9
South	534	23.0	15.6	7.4
Northeast	237	10.2	5.5	4.7
Midwest	134	5.8	2.8	2.9
North	43	1.9	0.9	1.0
Year of transplantation				
2000–2003	592	25.5	12.8	12.8
2004–2007	592	25.5	12.8	12.8
2008–2011	720	31.1	15.5	15.5
2012–2014	414	17.9	8.9	8.9
Recipient sex				
Female	852	36.8	18.4	18.4
Male	1466	63.2	31.6	31.6
Age group, years				
18–25	224	9.7	4.8	4.8
26–35	530	22.9	11.4	11.4
36–45	594	25.6	12.8	12.8
46–55	580	25.0	12.5	12.5
56–65	316	13.6	6.8	6.8
>65	74	3.2	1.6	1.6
Patient skin color ^a^				
White	312	55.0	23.1	31.9
Brown	178	31.4	23.1	18.7
Black	34	6.0	1.2	4.8
Others	43	7.6	3.9	3.7
Primary cause of chronic kidney disease				
Hypertension/cardiovascular disease	444	19.2	9.2	10.9
Nephritis ^b^	199	8.6	3.0	3.3
Organ failure or rejection	52	2.2	1.2	1.1
Diabetes mellitus	67	2.9	1.4	1.5
Kidney cystic disease/neoplasms /tumors	30	1.3	0.9	0.3
Uropathies	33	1.4	0.8	0.6
Infections/other causes/undetermined	1493	64.4	31.2	33.2
Donor type				
Living	1476	63.7	36.3	36.3
Deceased	842	36.3	18.2	18.2
Dialysis time before transplant, months ^a, c^				
≤26	1050	50.7	25.8	24.9
>26	1023	49.3	24.6	24.7
Events				
Censoring ^d^	1897	81.8	41.0	40.9
Graft loss	421	18.2	9.0	9.1
Death	320	13.8	7.2	6.6
Dialysis for more than 3 months	93	4.0	1.7	2.3
Re-transplant	8	0.4	0.2	0.2

^a^ Refers to individuals with valid data. ^b^ Glomerulonephritis/interstitial nephritis/pyelonephritis. ^c^ Median time = 26 months. ^d^ Lost to follow-up or right censoring (end of follow-up).

**Table 2:** Outcome measures of effectiveness of the study patients in a matched cohort (Brazil, 2000 to 2015; *n* = 2318).

**Variable**	**Graft Loss (%)**					
	**All Patients**		**Death**		**Dialysis for More than Three Months/Re-Transplant**	
	**421 (18.2%)**		**320 (13.8%)**		**101 (4.4%)**	
**Immunosuppressive Regimen**	**Cyclosporine Group**	**Tacrolimus Group**	**Cyclosporine Group**	**Tacrolimus Group**	**Cyclosporine Group**	**Tacrolimus Group**
Azathioprine	3.3	2.1	2.7	1.6	0.6	0.4
Mycophenolate	4.7	6.4	3.9	4.4	0.8	1.9
Monotherapy	0.7	0.4	0.5	0.3	0.2	0.1
Everolimus	0.1	0.0	0.1	0.0	0.0	0.0
Sirolimus	0.1	0.3	0.1	0.2	0.0	0.1
Other schemes	0.1	0.0	0.0	0.0	0.1	0.0
Total group	9.0	9.2	7.2	6.6	1.9	2.5
Hazard Ratio ^a^ (HR) Estimates for Graft Failure in Each Group Studied					**Survival ^b^ (95% Confidence Interval)**	
**Immunosuppressive Regimen**	**Cyclosporine Group**		**Tacrolimus Group**		**Cyclosporine Group**	**Tacrolimus Group**
	**HR (95% CI)**	***p***	**HR (95% CI)**	***p***		
Total					52.1 (44.7, 60.8)	47.5 (36.1, 62.7)
Azathioprine	0.75 (0.57, 0.99)	0.04	1.06 (0.78, 1.43)	0.68	54.0 (43.9, 66.4)	37.3 (21.5, 64.6)
Mycophenolate	1.30 (0.98, 1.71)	0.10	0.88 (0,65, 1,18)	0.39	60.9 (51.9, 71.5)	60.9 (52.1, 71.1)
Monotherapy	0.85 (0.51, 1.39)	0.50	1.28 (0.66, 2.47)	0.48	67.7 (54.8, 83.7)	51.4 (29.8, 88.9)
Everolimus ^c^	0.97 (0.24, 3.95)	0.97	NA		57.1 (24.3, 100.0) *	NA
Sirolimus	1.42 (0.35, 5.72)	0.62	2.02 (0.94, 4.18)	0.06	40,0 (29.7, 93.5) *	50.5 (28.0, 90.8) *

Note: ^a^ Result of the univariate analysis; ^b^ Survival graft survival rate at the end of 15 years of follow-up. NA: not possible to estimate, due to the small number of patients; ^c^ incorporated in SUS in 2008; * Regimen that did not have 15 years of observation.

**Table 3:** Annual graft survival rates of the study patients according to the calcineurin inhibitor group in a matched cohort (Brazil, 2000 to 2015; *n* = 2318).

Follow-Up Year	Graft Survival Rates (95% CI)		
	All Patients *n* = 2318	Cyclosporine Group *n* = 1159	Tacrolimus Group *n* = 1159
1st	99.2 (98.9, 99.6)	99.6 (99.2, 99.9)	98.9 (98.3, 99.5)
2nd	96.2 (95.4, 97.0)	96.7 (95.6, 97.8)	95.8 (94.6, 97.0)
3rd	93.5 (92.5, 94.6)	93.3 (91.8, 94.9)	93.8 (92.3, 95.2)
4th	90.4 (89.1, 91.7)	89.9 (88.0, 91.8)	90.9 (89.1, 92.7)
5th	87.4 (85.9, 88.9)	87.0 (84.8, 89.2)	87.3 (85.2, 89.6)
6th	83.5 (81.7, 85.3)	83.5 (80.9, 86.1)	83.5 (81.0, 86.1)
7th	79.9 (77.8, 82.0)	80.4 (77.5, 83.4)	79.4 (76.5, 82.5)
8th	74.9 (72.4, 77.5)	74.8 (71.2, 78.5)	75.0 (71.6, 78.6)
9th	71.4 (68.6, 74.3)	70.4 (66.3, 74.7)	71.9 (68.1, 75.9)
10th	67.2 (64.0, 70.6)	66.7 (62.1, 71.6)	67.7 (63.2, 72.5)
11th	64.1 (60.5, 68.0)	61.8 (56.4, 67.7)	64.7 (59.0, 69.7)
12th	59.1 (54.9, 63.7)	59.1 (53.2, 65.6)	57.1 (50.9, 64.2)
13th	55.1 (50.3, 60.3)	56.6 (50.2, 63.9)	56.0 (49.6, 63.3)
14th	52.9 (47.4, 58.9)	54.1 (47.2, 62.0)	53.5 (45.9, 62.3)
15th	50.1 (43.0, 58.3)	52.1 (44.7, 60.8)	47.5 (36.1, 62.7)

CI: confidence interval.

**Table 4:** Hazard ratios for graft loss according to Cox logistic regression of a 15-year follow-up (Brazil 2000–2015; *n* = 2318).

Variable	HR (95% CI)	*p*-Value
Primary cause of chronic kidney disease		
Diabetes mellitus	2.55 (1.55, 3.71)	<0.01
Hypertension/cardiovascular diseases	1.28 (1.02, 1.61)	0.033
Dialysis length of time before transplant (>26 months)	1.53 (1.25, 1.88)	<0.01

HR: hazard ratio; CI: confidence interval.

**Table 5:** Hazard ratios for graft loss according to Cox logistic regression of a 15-year follow-up considering patient race/color (Brazil, 2000–2015; *n* = 567).

Variable	HR (95% CI)	*p* Value
Race/color of the patient		
Black	2.61 (1.13, 6.02)	0.024
Primary cause of chronic kidney disease		
Diabetes Mellitus	3.14 (1.11, 8.81)	0.029
Therapeutic regimen		
Cyclosporine + Azathioprine	1.0	
Cyclosporine	1.35 (0.37, 4.92)	0.647
Cyclosporine + Mycophenolate	2.14 (0.97, 4.67)	0.058
Cyclosporine + Sirolimus	NA	
Cyclosporine + Everolimus	NA	
Tacrolimus + Azathioprine	1.02 ( 0.40, 2.56)	0.967
Tacrolimus	3.93 (0.86, 18.05)	0.077
Tacrolimus + Mycophenolate	2.17 (1.02, 2.41)	0.028
Tacrolimus + Sirolimus	4.63 (1.007, 21.26)	0.050
Tacrolimus + Everolimus	NA	

NA: not possible to estimate due to small numbers; HR: hazard ratio; CI: confidence interval.
